# chTLR4 pathway activation by *Astragalus* polysaccharide in bursa of Fabricius

**DOI:** 10.1186/s12917-017-1039-y

**Published:** 2017-05-02

**Authors:** Zhang Ruili, Yu Qun, Shi Guangliang, Liu Rui, Zhang Weiqian, Zhao Xia, Li Guangxing, Ge Ming

**Affiliations:** 1 College of Veterinary Medicine, Northeast Agricultural University, Harbin, 150030 China; 2 Harbin Veterinary Research Institute, Chinese Academy Agricultural Sciences, Harbin, 150001 China

**Keywords:** *Astragalus* polysaccharide, Bursa of Fabricius, Innate immunity, Toll-like receptor 4

## Abstract

**Background:**

The Toll-like receptor 4 (TLR4) pathway involves in the pathogen recognition and defense against infection in mammals. Considering that avian and mammalian TLR are differentially mediated, the action of a natural product on avian TLR4 pathway was unclear. High, medium and low doses of *Astragalus* polysaccharide (APS), were treated the chicken at 7-days-old age by gavage. The sIgA level in the intestinal fluid, the expression of chTLR4 mRNA/protein in bursa of Fabricius as well as the expression of downstream molecules of chTLR4 (chMyD88, chTRIF, chNF-κB, chIRF3, chIFN-β and chTNF-α) were measured on alternate days.

**Results:**

The content of sIgA and the chTLR4 mRNA expression/protein level were increased in non-dose-dependent manner after APS supplement. Also, the expressions of a subset of MyD88-independent pathway genes were more than MyD88-independent, in particular with low doses of APS supplement for 7 days.

**Conclusions:**

These suggest that administration of APS activates chTLR4 pathway in bursa of Fabricius in MyD88-independent pathway. Meanwhile, low dose of APS shows better performance regarding the activation of chTLR4 and regulation of MyD88-independent pathway.

## Background

*Astragalus* polysaccharide (APS), a medicinal herb, has been used for thousands years in China. Numerous studies have demonstrated that APS has a wide range of antiviral, antioxidant, anti-inflammatory, and immunoregulatory properties in mammals [1–4]. Shao et al. demonstrated for the first time that APS directly interacted with Toll-like receptor 4 (TLR4) molecule [2]. Toll-like receptor family (TLRs) as important components of innate immune response can bind to specific antigen, activate a signal transduction pathway and accelerate the release of inflammatory cytokines in early pathogen recognition, which favors the initiation of specific adaptive immune response [2, 5]. Recent scientific investigations conducted in the models of porcine reproductive and respiratory syndrome virus and H9N2 avian influenza virus have reported APS could exert immunoprotecting effect [4, 6]. However, these are confined to inflammatory cytokines instead of pathway. Understanding cell signaling networks that underlie metabolic pathway helps unravel the nature of the innate immune response. Despite the relationship between APS and murine TLR4 molecule has been clarified, avian and mammalian TLR may be differentially adapted to pathogen-derived ligand recognition [5, 7]. As such, this study is to use bursa of Fabricus for investigating the potential mechanism of APS targeting TLR4 pathway.

## Methods

### Animals and treatment

A total of 80 1-day-old Hyline brown chickens were purchased from Harbin Veterinary Research Institute, China. These chickens were accommodated and feeded at Northeast Agricultural University according to the Animal Welfare Protocol (Harbin, China, #NEAU-2013-02-0252-11). At 7 days-age-old, chickens were randomly divided into four groups: the high-dose group of APS (APS_H_: 80 mg/kg), medium-dose group (APS_M_: 40 mg/kg), low-dose group (APS_L_: 20 mg/kg) and control group (an equivalent volume of saline), and all chickens were treated by gavage for 7 days. The APS were extracted by the Sevag method according to our previous study [8]. After alternate days of treatment, i.e., on days 1, 3, 5, and 7, 5 chickens in per group were randomly selected for euthanasia and then bursa of Fabricius were collected and placed on the ice.

### The content of secretory immunoglobulin a (sIgA) in intestinal fluid

The content of sIgA was detected by the indirect ELISA method. Intestinal fluid, diluted 1:20 with 0.05 M carbonate buffer (pH 9.6) was added to 96-well polystyrene immunoplates for overnight at 4 °C. The plates were incubated with a 1:1500 dilution of goat-anti-chicken-IgA monoclonal antibody (Abcam, Cambridge, MA, USA) and 1:5000 horseradish peroxidase conjugated goat-anti-rabbit-IgG (Wuhan Boster Biological Technology LTD., China), respectively. After exposure of the wells with 4-chlorine-1-naphthol, absorbance values were measured in Microplate Reader at 490 nm (Gemini EM, CA, USA).

### The mRNA expression in bursa of Fabricius

Total RNA was extracted from the chicken bursa of Fabricius according to Trizol’s protocol and then was reverse transcribed into cDNA. Specific primers were designed according to the sequences of the chicken TLR4 (chTLR4), MyD88 (chMyD88), TRIF (chTRIF), NF-κB (chNF-κB), IRF3 (chIRF3), IFN-β (chIFN-β), TNF-α (chTNF-α) genes provided by GenBank (Table 1). Real-time fluorescence quantitative PCR was performed according to the protocol (ABI Prism®7500, MA, USA). The results were analyzed using the 2^–ΔΔCt^ method with chβ-actin as an internal reference.

**Table 1:** Primer design

Names of primers	Sequences(5′-3′)	Product length	Accession no.
chTLR4	TTCCAAGCACCAGATAGCAACATC	202	NM_001030693.1
	ACGGGTCACAGAAGAACTTAGGG		
chMyD88	AGCGTGCCAAAGACTTCAGA	251	NM_001030962.3
	ACCATCCTCCGACACCTTCT		
chTRIF	AGCCTGATGGAGAGAGACAGAG	139	NM_001081506.1
	GATAGACGAGAGGAACTGACCTG		
chNF-κB	TCTGAACAGCAAGTCATCCATAACG	255	NM_205134.1
	AAGGAAGTGAGGTTGAGGAGTCG		
chIRF3	CTCTCTGACTCTTTCAACCTCTTCG	260	NM_205372.1
	TGCTGCCTGCTCCTGTGG		

### The expression of chTLR4 protein in bursa of Fabricius

The chicken bursa of Fabricius was fixed in 4% formaldehyde, sliced into paraffin sections and deparaffinized. The sections were blocked with 1:20 goat serum and incubated with rabbit-anti-chicken-chTLR4 multi-clonal antibody (made by our group) for overnight at 4 °C, following incubation with 1:200 horseradish peroxidase-conjugated goat anti-rabbit. Protein expression was visualized by staining with 3,3′-diaminobenzidine, and cells were stained by hematoxylin. At 12- days-old- age, chicken were intravenously injected with lipopolysaccharide (LPS) at a dose of 1 mg/kg at 24 h before bursa of Fabricus collection. As TLR4 that senses LPS resulting in the increase of TLR4-immunopositive cells, LPS group was regarded as a positive control group. Positive expression appeared brownish yellow granules under light microscopy.

### Statistic

Date are represented as the Mean ± SEM and analyzed by two-way ANOVA. Fisher’s LSD test was performed post hoc (SPSS 19.0, USA). * *p* < 0.05, ** *p* < 0.01 and *** *p* < 0.001 are shown as statistically significant.

## Results and discussion

TLR4 is a part of TLRs—evolutionarily conserved pattern recognition receptors, which involved in the innate defense against infection. Several evidences suggest that TLR4 is a receptor of LPS in mammals. Chickens have also evolved the resistant to LPS infection [9, 10]. Keestra et al. have reported that TLR4 was activated in response to LPS via the MyD88-dependent pathway in chickens [9].

Apart from LPS, various polysaccharides found in medicinal herbs such as APS, *Angelica sinensis* polysaccharide, *Epimedium* polysaccharides, and *Lycium barbarum* polysaccharides, are also able to activate macrophages through TLR 4-dependent pathway [11–13]. Compared with LPS, APS has attracted more attentions because of its nontoxic and lower cost. Whether APS could likewise activate chTLR4-mediated MyD88-dependent pathway remains unclear. Thus, the present study was conducted to investigate this.

### Addition of APS increases sIgA contents non-dose-dependently in intestinal fluid

The defense of the intestinal mucosal immunity against pathogens is mediated initially by sIgA. Its binding to antigen interferes with pathogen attachment and colonization. These occur particularly without maternal antibodies, which would be depleted by 10 days of age in chicken [14]. In our study, we supplied APS for 7-days-old chicken to investigate its effect on chTLR4 pathway during early mature stage. Nonetheless, maternal antibodies’ influence should been considered after administration of APS. Accordingly, sIgA is used to indicate the depletion of maternal antibodies for early maturation in chicken.

**Fig. 1:**
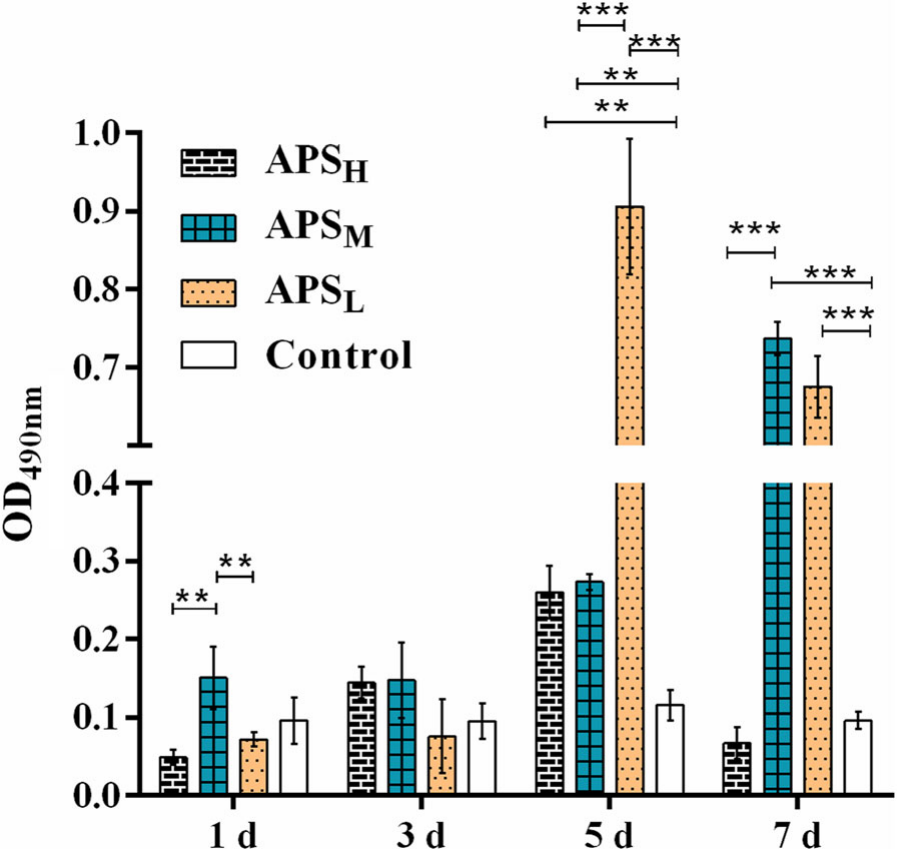
The change of sIgA contents in chicken intestine fluid. Chickens were exposed to various doses (APS_H_: 80 mg/kg, APS_M_: 40 mg/kg, APS_L_: 20 mg/kg and normal saline by gavage once a day for 5 days at 7 days of age. Intestine fluid was collected after treatment 1, 3, 5 or 7 days, and sIgA content was measured by indirect ELISA. Results represent the means ± SEM of values for three chickens per group. * *p* < 0.05 and *** *p* < 0.001 are considered as significant difference

For 8-days-old chicken, the changes of sIgA levels varied by the dose of APS supplement. Although the increase in sIgA levels was observed after the addition of APS_M_ compared to APS_L_ and APS_H_, these additions could not have statistically significance (Fig. 1). Before 10-days of age, APS could not also show significant influence on content of sIgA compared with control group, whereas their levels were increased in the chicken intestinal fluid after 5 days of APS supplement, except 7 days of APS_H_ supplement. APS was able to bind to sIgA with intermediate to high affinity and this binding also induced the proliferation [2]. These indicate the presence of maternal antibodies prevented from APS binding to sIgA. In contrast, the depletion of maternal antibodies improved their bindings. Furthermore, the administration of APS_H_ was likely to competitively suppress this binding after 1 week’s treatment. APS_L_ had better performance than APS_M_. Therefore, addition of APS increased sIgA contents non-dose-dependently in intestinal fluid.

### Administration of APS improves chTLR4 mRNA and protein expression in a time dependent manner in bursa of Fabricius

Keestra et al. reported that chickens sensed LPS via TLR4 rather than chicken TLR2 receptor [9]. To determine whether chTLR4 is activated after APS treatment, we tested the expressions of TLRs mRNA in pilot study. Our data showed the increase of chTLR4 mRNA expression was stronger than others in the presence of APS (not shown).

**Fig. 2:**
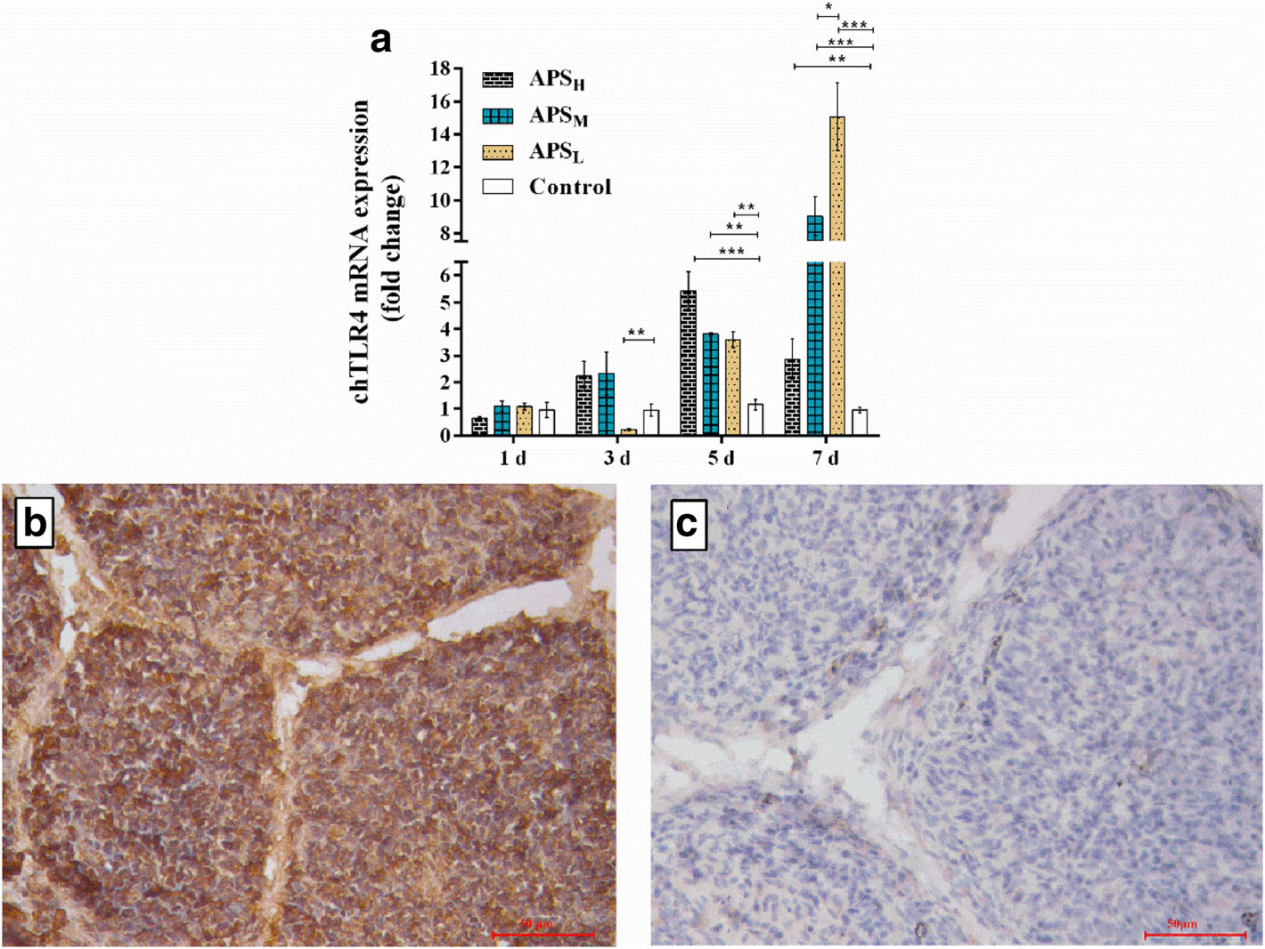
chTLR4 mRNA and protein expression in bursa of Fabricius. (**a**): Dynamic change of mRNA expression of chTLR4 in chicken bursa of Fabricius was determined by qPCR after treatment by gavage for 7 days with or without various dose of APS (APS_H_:80 mg/kg, APS_M_:40 mg/kg, APS_L_:20 mg/kg and normal saline). The chTLR4 mRNA gene expression was normalized to β-actin expression, and standardized to 1.0 in control group. Protein expression of chTLR4 in chicken bursa of Fabricius with the administration of APS_L_ after 5 days was shown in (**b**). chTLR4 protein expression was observed in the chicken bursa of Fabricius follicular cortex and medulla cells by immunohistochemical staining for three independent samples (**b**), and (**c**) treated without rabbit-anti-chicken-chTLR4 (multi-clonal antibody made by our lab) is negative stain. Results represent the means ± SEM of values for five chickens per group.* *p* < 0.05, ** *p* < 0.01 and *** *p* < 0.001 is considered as significant difference

In this study, chTLR4 mRNA and protein expression within the bursa of Fabricius were measured at different times post-treatment. As shown in Fig. 2a, chTLR4 mRNA expression levels were increased significantly after 5 and 7 days of APS addition. And thus APS addition increased chTLR4 mRNA expression in a time dependent manner in bursa of Fabricius. As for various doses of APS addition, the levels of chTLR4 mRNA expression following treatment with APS_L_ fluctuated with time. In the first day, its level was higher than control group, but there was not shown significantly between them. Over the next 2 days, the concentration of chTLR4 mRNA dropped to 0.21 folds. Following 4 days, its content rose sharply, especially up to 15.3 folds after 7 days. Unlike the U-shaped trend of APS_L_, APS_M_ and APS_H_ increased chTLR mRNA express in time-dependent manner and reached their peaks at 9.03 and 5.42 times after 7 and 5 days, respectively. As a result, administration of APS_L_ with higher growth rate is used practicably in the poultry industry, regardless of the duration treatment. Besides, the previous study has pointed out the low dose of LPS can stimulate TLR4 expression and strengthen the immune system, rather than large doses of LPS [15]. These results were in accordance with sIgA as shown above. Based on these considerations, we employed immunohistochemistry to only test protein expression after 5 days of APS_L_ addition.

Figure 2b and c showed the changes of chTLR4 protein expression in bursa of Fabricius after treatment of APS. chTLR4 protein was found the wide expression in bursa of Fabricius lymphoid follicle cortex and medulla cells after the addition of APS, suggesting that APS had a capability of improving chTLR4 protein expression.

### Supplement of APS enhances the expression of a subset of the chMyD88-independent-inducible genes in bursa of Fabricius

**Fig. 3:**
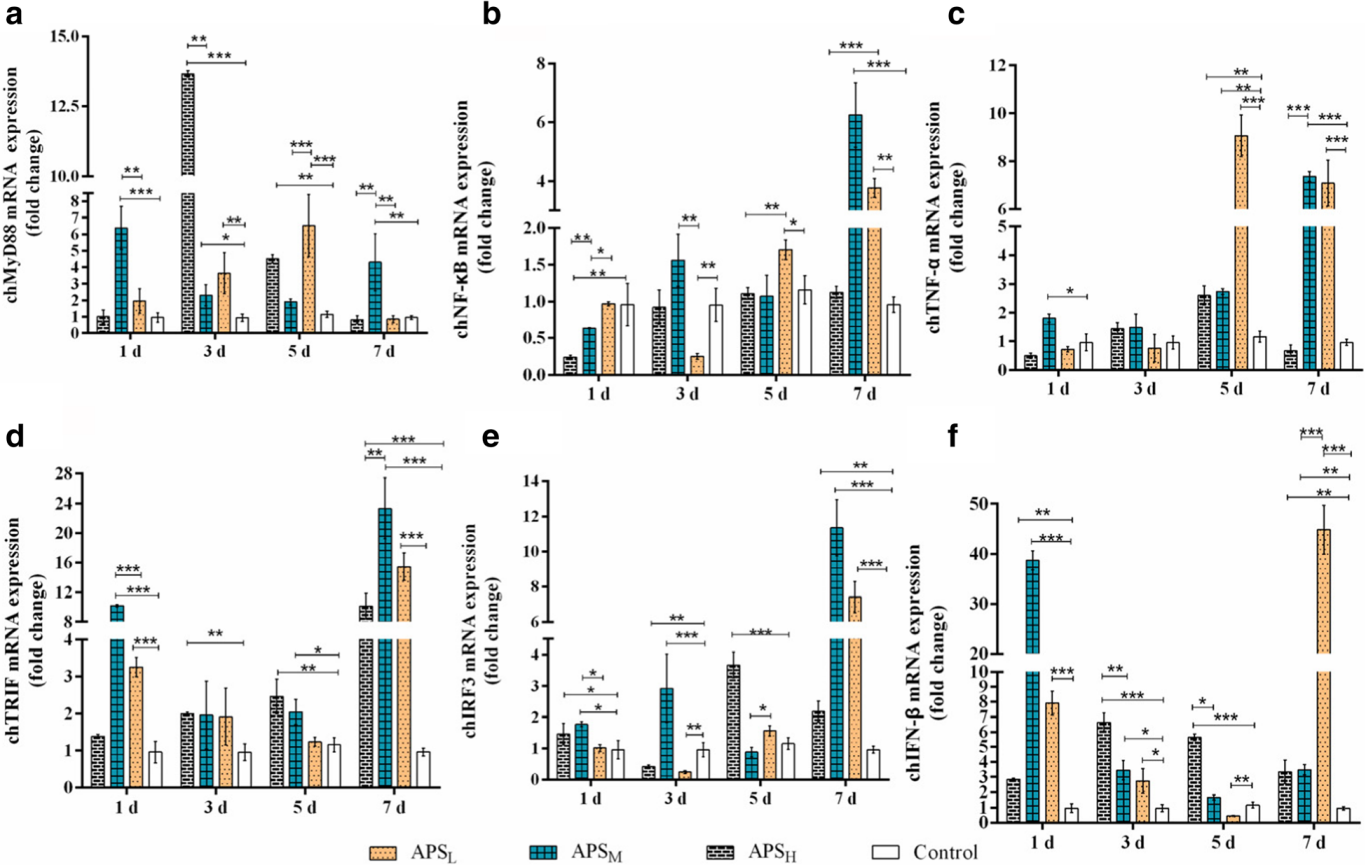
The changes in the gene expression of the chTLR4 signal pathway genes in the bursa of Fabricius. The expression of chMyD88 (**a**), chNF-κB (**b**), chTNF-α (**c**), chTRIF (**d**), chIFNβ (**e**) and chIRF3 (**f**) under chTLR4 signal pathway was determined by qPCR. Results represent the means ± SEM of values normalized to β-actin expression and standardized to 1.0 in control group. Results represent the means ± SEM of values for five chickens per group.* *p* < 0.05, ** *p* < 0.01 and *** *p* < 0.001 is considered as significant difference

TLR4 could be stimulated by two pathways: the MyD88-dependent and TRIF-dependent (MyD88-independent) pathways. To better understand of molecular targets of APS, we assessed the activity of both signaling routes in chicken and measured the mRNA levels for downstream molecules of chTLR4, including chMyD88, chTRIF, chNF-κB, chIRF3, chIFN-β and chTNF-α. Our results showed that APS upregulated both two pathways (Fig. 3). Activation of the MyD88-dependent pathway enhanced transcription of a number of NF-κB regulated genes, which leads to the production of inflammatory cytokines, including TNF-α. Stimulation of the TRIF-dependent pathway activates IRF3, which leads to the production of IFN-β [2, 9]. From the point of total fold changes, the expressions of a subset of MyD88-independent pathway genes were more than MyD88-dependent pathway, in particular with APS_L_ supplement after 7 days. There was concurrent promotion of TRIF, IRF3 and IFN-β mRNA expression (Fig. 3d-e), suggesting TRIF-dependent pathway was intact in response to APS; on the contrary, chMyD88, chNF-κB and chTNF-α show divergent changes with various doses of APS (Fig. 3a-c). As a result, supplement of APS enhanced the expression of a subset of the chMyD88-independent-inducible genes in bursa of Fabricius.

Interestingly, APS_H_ treatment induced a rapid induction of chMyD88 expression, whereas APS_M_ induced a gradual and delayed induction (Fig. 3a). We assumed that this might be related with the non-unique activation of TLR4 molecules in the presence of APS [1]. Also, chMyD88-dependent pathway could be stimulated by all TLRs. As a consequence of existence of other chTLR activation, for example chTLR2, chMyD88 expression were shown irregular pattern within various doses of APS. Conversely, the TRIF-dependent pathway is unique to TLR3 and TLR4 signaling pathway. Therefore, our data also indicate APS treatment might not interact with chTLR3. It is noteworthy that the expression of chNF-κB mRNA was the lowest among downstream molecules (Fig. 3b). These suggest that chNF-κB might be involved in two pathways with APS addition. Furthermore, the present or absence of maternal antibodies may cause a biphasic increase in chTRIF and chIFN-β after 1 and 7 days. To date, the mechanism of action of APS has been not fully defined. There are further questions to address in the future.

## Conclusions

APS_L_ supplement activates chTLR4 within bursa of Fabricius in non-dose-dependent manner; in addition, it increases the expression of chMyD88-indepent-induciable genes among the downstream molecules of chTLR4. The action of APS was not shown time-dependent in 7-days-old chicken, which might be in relation to maternal antibodies.

## Abbreviations

APS: *Astragalus* polysaccharide
chTLR4: Chicken toll-like receptor 4

## References

[1] Shi L, Yin F, Xin X, Mao S, Hu P, Zhao C, Sun X (2014). Astragalus polysaccharide protects Astrocytes from being infected by HSV-1 through TLR3/NF-κB Signaling pathway. Evid Based Complement Alternat Med.

[2] Shao B-M, Xu W, Dai H, Tu P, Li Z, Gao X-M (2004). A study on the immune receptors for polysaccharides from the roots of Astragalus Membranaceus, a Chinese medicinal herb. Biochem Bioph Res C.

[3] Dai H, Jia G, Liu X, Liu Z, Wang H (2014). Astragalus polysaccharide inhibits isoprenaline-induced cardiac hypertrophy via suppressing ca(2)(+)-mediated calcineurin/NFATc3 and CaMKII signaling cascades. Environ Toxicol Pharmacol.

[4] Zhuge ZY, Zhu YH, Liu PQ, Yan XD, Yue Y, Weng XG, Zhang R, Wang JF (2012). Effects of Astragalus polysaccharide on immune responses of porcine PBMC stimulated with PRRSV or CSFV. PLoS One.

[5] de Zoete MR, Keestra AM, Roszczenko P, van Putten JPM (2010). Activation of human and chicken toll-like receptors by campylobacter spp. Infect Immun.

[6] Kallon S, Li X, Ji J, Chen C, Xi Q, Chang S, Xue C, Ma J, Xie Q, Zhang Y (2013). Astragalus polysaccharide enhances immunity and inhibits H9N2 avian influenza virus in vitro and in vivo. J Anim Sci Biotechnol.

[7] Vinkler M, Bainova H, Bryja J (2014). Protein evolution of toll-like receptors 4, 5 and 7 within Galloanserae birds. Genet Sel Evol.

[8] Ge M, Zhang W, Shi G, Xiao C, Zhao X, Zhang R (2015). Astragalus polysaccharide perseveres Cytomembrane capacity against Newcastle disease virus infection. Pak Vet J.

[9] Keestra AM, van Putten JPM (2008). Unique properties of the chicken TLR4/MD-2 complex: selective Lipopolysaccharide activation of the MyD88-dependent pathway. J Immunol.

[10] Zhang Y, Guo F, Ni Y, Zhao R (2013). LPS-induced inflammation in the chicken is associated with CCAAT/enhancer binding protein beta-mediated fat mass and obesity associated gene down-regulation in the liver but not hypothalamus. BMC Vet Res.

[11] Guo L, Wang D, Hu Y, Zhao X, Wang Y, Yang S, Wang J, Fan Y, Han G, Gao H (2012). Adjuvanticity of compound polysaccharides on chickens against Newcastle disease and avian influenza vaccine. Int J Biol Macromol.

[12] Yang T, Jia M, Zhou S, Pan F, Mei Q (2012). Antivirus and immune enhancement activities of sulfated polysaccharide from Angelica Sinensis. Int J Biol Macromol.

[13] Su C-x, Duan X-g, Liang L-j, Feng W, Zheng J, Fu X-Y, Yan Y-m, Ling H, Wang N-p (2014). *Lycium barbarum* Polysaccharides as an adjuvant for recombinant vaccine through enhancement of humoral immunity by activating Tfh cells. Vet Immunol Immuopathol.

[14] Gharaibeh S, Mahmoud K (2013). Decay of maternal antibodies in broiler chickens. Poult Sci.

[15] Hsu H-Y, Hua K-F, Lin C-C, Lin C-H, Hsu J, Wong C-H (2004). Extract of Reishi polysaccharides induces cytokine expression via TLR4-modulated protein Kinase Signaling pathways. J Immunol.

