# Erratum to: chTLR4 pathway activation by *Astragalus* polysaccharide in bursa of Fabricius

**DOI:** 10.1186/s12917-017-1049-9

**Published:** 2017-05-10

**Authors:** Ruili Zhang, Qun Yu, Guangliang Shi, Rui Liu, Weiqian Zhang, Xia Zhao, Guangxing Li, Ming Ge

**Affiliations:** 1 College of Veterinary Medicine, Northeast Agricultural University, Harbin, 150030 China; 2 Harbin Veterinary Research Institute, Chinese Academy Agricultural Sciences, Harbin, 150001 China

## Erratum

The original article [1] contains an error whereby the authors’ given & family names were mistakenly interchanged.

The original article has now been corrected to present the authors’ names correctly.
